# Supplementary material for: VezA/vezatin facilitates proper assembly of the dynactin complex in vivo
Source: bioRxiv. 2024 Apr 20:2024.04.19.590248. Preprint. [Version 1] doi: 10.1101/2024.04.19.590248 (PMC11042379; doi:10.1101/2024.04.19.590248)
Supplement: 1 [file NIHPP2024.04.19.590248V1-supplement-1.pdf]

bioRxiv preprint doi: https://doi.org/10.1101/2024.04.19.590248; this version posted April 20, 2024. The copyright holder for this preprint (which was not certified by peer review) is the author/funder. This article is a US Government work. It is not subject to copyright under 17 USC 105 and is also made available for use under a CC0 license.

Figure S1. An AlphaFold2-based analysis of VezA-dynactin interaction. (A) An AlphaFold2 prediction model of the VezA dimer binding to Arp1 as well as the pointed end. In this prediction, we included four copies of Arp1 but no convention actin, although conventional actin is located next to Arp11 in the structure of vertebrate dynactin (Urnavicius et al., 2015). This is because our pulldown data were not able to provide evidence that conventional actin is a component of dynactin in *A. nidulans* (Zhang et al., 2018). (B) In this model, the N-terminus of VezA ($VezA^{1-20}$) is close to p25 and p62 of the pointed end. (C) In the same model, the C-terminus of VezA ($VezA^{563-615}$) is close to the Arp1 mini-filament. (D) A western blot showing the protein levels of VezA-GFP in the *vezA*$^{1-615}$-GFP (full-length), *vezA*$^{\Delta 1-20}$-GFP and *vezA*$^{\Delta 563-615}$-GFP strains. (E) A quantitative analysis on the effects of the *vezA*$^{\Delta 1-20}$ and *vezA*$^{\Delta 563-615}$ mutations on the level of VezA-GFP. The values were generated from western blot analyses of three independent pull-down experiments (*n* = 3 for all). Scatterplots with mean and SD values as well as p values were generated by Prism 10 (ordinary one-way ANOVA test with Dunnett's multiple comparisons test). (F) Microscopic images showing the distributions of mCherry-RabA-labeled early endosomes in the *vezA*$^{\Delta 1-20}$ and *vezA*$^{\Delta 563-615}$ mutants. Hyphal tip is indicated by a yellow arrowhead. Bar, 10 μm. (G) A quantitative analysis on the percentage of hyphal tips with the abnormal accumulation of early endosomes. Three experiments were performed, and in each experiment, 50 or more hyphal tips were examined for each strain. Scatterplots with mean and SD values as well as p values were generated by Prism 10 (ordinary one-way ANOVA test with Tukey's multiple comparisons test).

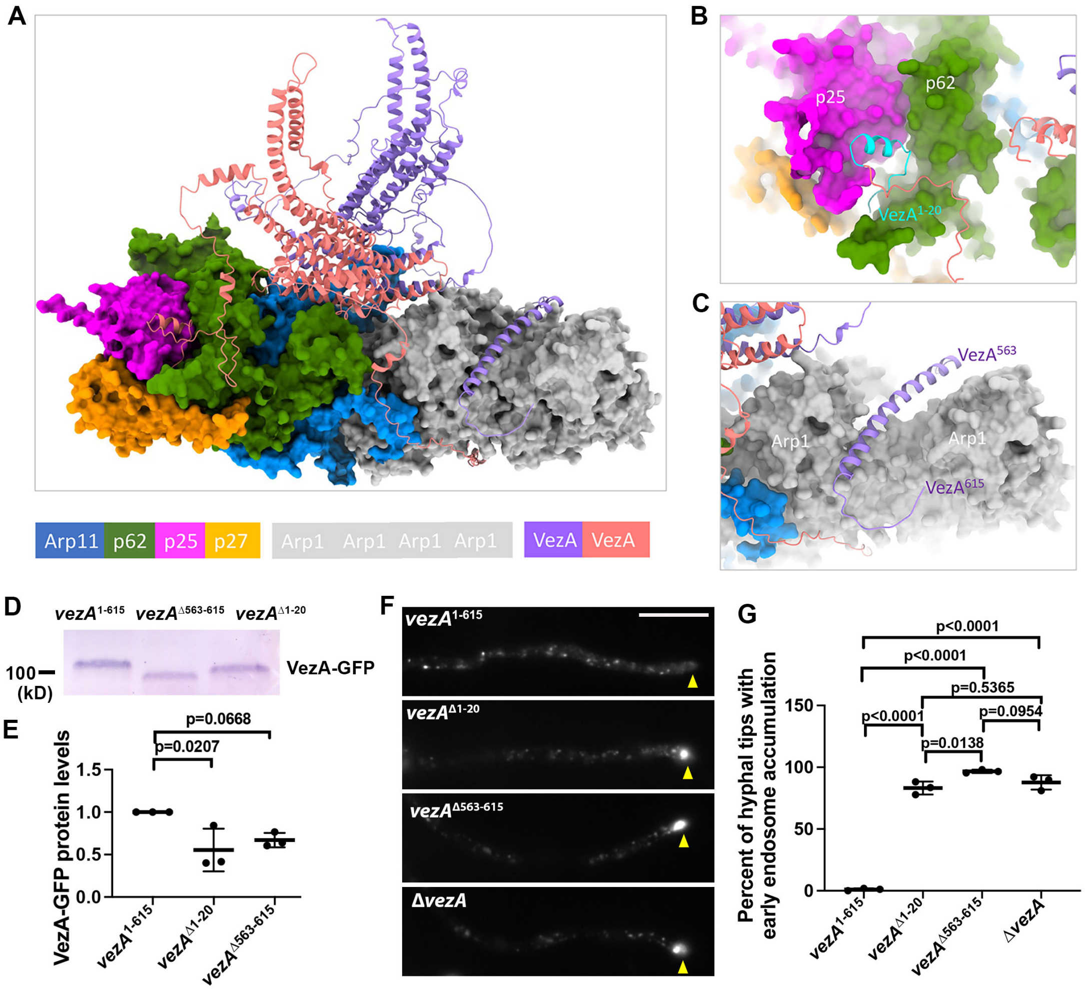

bioRxiv preprint doi: https://doi.org/10.1101/2024.04.19.590248; this version posted April 20, 2024. The copyright holder for this preprint (which was not certified by peer review) is the author/funder. This article is a US Government work. It is not subject to copyright under 17 USC 105 and is also made available for use under a CC0 license.

Figure S2. VezA affects the microtubule plus-end localization of several pointed-end proteins. (A) Images of Arp11-GFP, p62-GFP and p25-GFP accumulation at the microtubule plus ends as represented by comets near the hyphal tip in the wild-type and Δ*vezA* strains. Hyphal tip is indicated by a yellow arrowhead. Bar, 5 μm. (B) Images of Arp11-GFP, p62-GFP and p25-GFP in the Δ*hookA* single mutant and the Δ*hookA*, Δ*vezA* double mutant strains. Hyphal tip is indicated by a yellow arrowhead. Bar, 5 μm. (C) Quantitative analyses on plus-end comet intensity of Arp11-GFP, p62-GFP and p25-GFP in the Δ*hookA* and Δ*hookA*, Δ*vezA* strains. The average value for the intensity in the Δ*hookA* single mutant is set as 1. Scatter plots with mean and S.D. values were generated by Prism 10, and the p values were generated by Mann-Whitney test (unpaired).

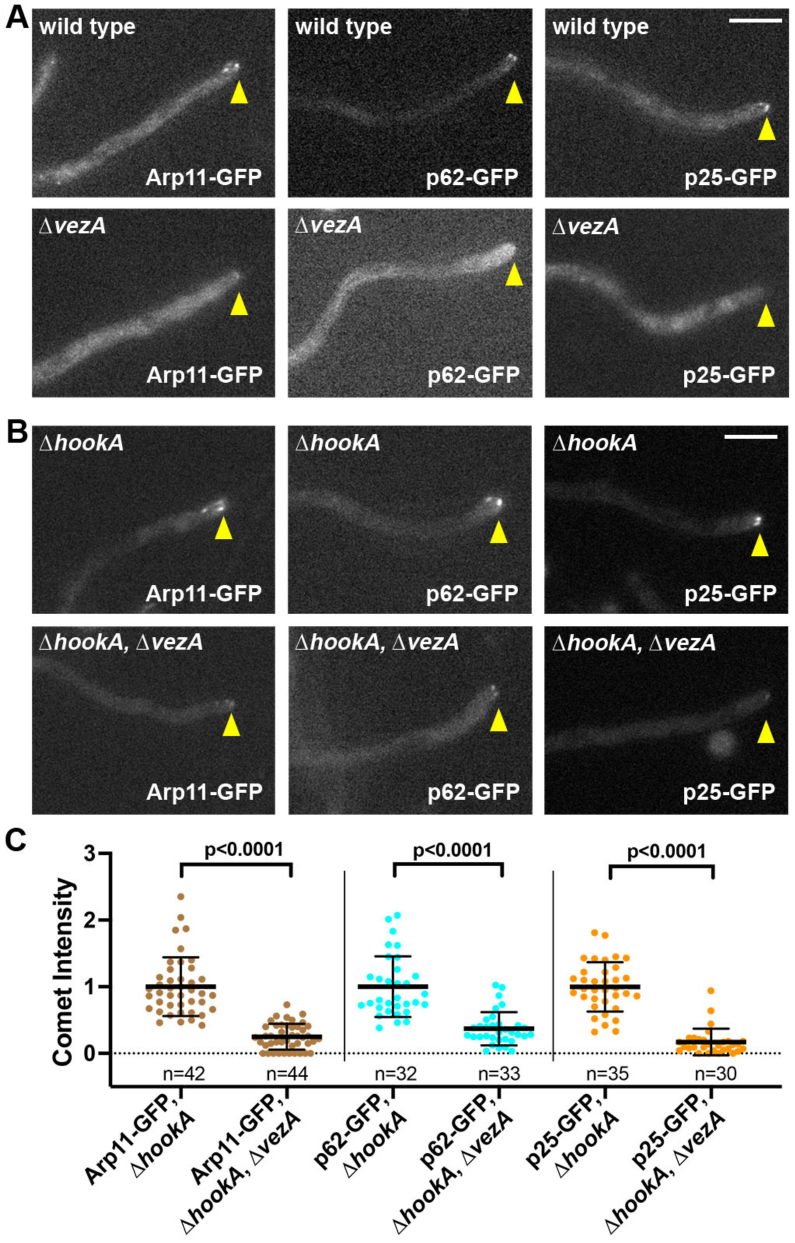

bioRxiv preprint doi: https://doi.org/10.1101/2024.04.19.590248; this version posted April 20, 2024. The copyright holder for this preprint (which was not certified by peer review) is the author/funder. This article is a US Government work. It is not subject to copyright under 17 USC 105 and is also made available for use under a CC0 license.

Figure S3. Nuclear distribution is defective in the Δ*vezA* mutant. (A) Histone H1-GFP-labeled nuclei in wild type and the Δ*vezA* mutant. Strains were grown overnight at 32°C in liquid minimal medium containing 1% glycerol. Yellow arrow head indicates the spore swelling. Bar, 5 μm. (B) A quantitative analysis on the percent of germ tubes containing different numbers of nuclei in the spore swelling. Column bar graphs with mean and S.D. values were generated from five experiments. For each experiment, at least 36 germ tubes were counted from each strain, and the total numbers of counted germ tubes are 229 for the wild-type control and 238 for the Δ*vezA* mutant. P values were generated from two-way ANOVA with Bonferroni's multiple comparisons test. (C) A quantitative analysis on the distance from the hyphal tip to the nucleus closest to it. Scatterplots with mean and SD values as well as p values were generated by Prism 10 (Student's *t* test, two tailed, unpaired).

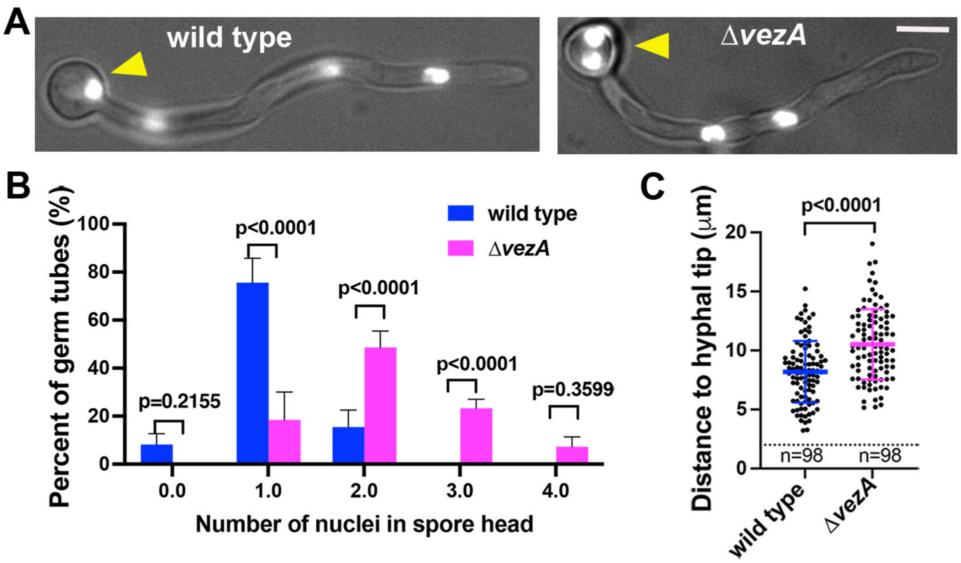

bioRxiv preprint doi: https://doi.org/10.1101/2024.04.19.590248; this version posted April 20, 2024. The copyright holder for this preprint (which was not certified by peer review) is the author/funder. This article is a US Government work. It is not subject to copyright under 17 USC 105 and is also made available for use under a CC0 license.

Table S1 Mass spectrometry data showing numbers of total peptides and unique peptides (in parentheses) of proteins pulled down with ΔTM-VezA-GFP in the wild-type background (Control) and in the *alcA*-Arp11, *alcA*-p50 or *alcA*-Arp1 background where expression of the *alcA*-promoter-controlled gene is repressed by glucose.

Protein/ numbers of total and unique peptides (unique peptides in parentheses)	**Control**	***alcA*-Arp11**	***alcA*-p50**	***alcA*-Arp1**	***alcA*-Arp11/ control** (ratio of total peptides)	***alcA*-p50/ control** (ratio of total peptides)	***alcA*-Arp1/ control** (ratio of total peptides)
**Dynein HC** (An0118, 4345 aa)	199 (183)	13 (13)	17 (17)	7 (6)	0.07	0.09	0.04
**p150** of dynactin (An6323, 1342 aa)	59 (57)	3 (3)	0 (0)	0 (0)	0.05	0	0
**p50** of dynactin (An3589, 467 aa)	24 (23)	2 (2)	1 (1)	0 (0)	0.08	0.04	0
**Arp1** of dynactin (An1953, 380 aa)	30 (25)	5 (5)	19 (17)	2 (2)	0.17	0.63	0.07
**Arp11** of dynactin (An3185, 557 aa)	34 (29)	1 (1)	31 (26)	12 (11)	0.03	0.91	0.35
**p62** of dynactin (An4917, 637 aa)	18 (17)	1 (1)	22 (21)	4 (4)	0.06	1.22	0.22
**p25** of dynactin (An5022, 202 aa)	6 (6)	0 (0)	6 (6)	3 (3)	0	1.00	0.50
**GFP** (238 aa)	29 (17)	28 (12)	29 (16)	27 (12)	0.97	1.00	0.93
**Myosin V** (An8862, 1569aa)	105 (94)	106 (75)	106 (84)	106 (85)	1.01	1.01	1.01
**NudF/LIS1** (An6197, 444aa)	18 (16)	3 (3)	2 (2)	0 (0)	0.17	0.11	0

Note that ΔTM-VezA-GFP also pulled down Myosin V but the amount of pulled-down Myosin V is not affected by the loss of Arp11 (*alcA*-Arp11), p50 (*alcA*-p50) or Arp1 (*alcA*-Arp1).

bioRxiv preprint doi: https://doi.org/10.1101/2024.04.19.590248; this version posted April 20, 2024. The copyright holder for this preprint (which was not certified by peer review) is the author/funder. This article is a US Government work. It is not subject to copyright under 17 USC 105 and is also made available for use under a CC0 license.

Table S2. Mass spectrometry data from three experiments showing numbers of total peptides and unique peptides of proteins pulled down with Arp11-GFP in the wild-type background (*vezA*+) and in the Δ*vezA* background.

Protein/ number of peptides (Experiment 1)	*vezA*+ (unique)	***vezA*+ (total)**	Δ*vezA* (unique)	**Δ*vezA* (total)**	**Δ*vezA*/*vezA*+ (ratio of total peptides relative to the Arp11 ratio set as 1)**
**p150** (An6323, 1342 aa)	40	**78**	12	**19**	**0.24**
**p50** (An3589, 467 aa)	21	**67**	10	**14**	**0.21**
**Arp1** (An1953, 380 aa)	12	**79**	5	**13**	**0.13**
**Arp11** (An3185, 557 aa)	17	**51**	19	**66**	**1**
**p62** (An4917, 637 aa)	12	**24**	9	**26**	**0.84**
**p25** (An5022, 202 aa)	4	**12**	5	**24**	**1.55**
**Cap alpha** (An2126, 273 aa)	6	**13**	4	**6**	**0.36**
**Cap beta** (An0290, 266aa)	4	**8**	0	**0**	**0**
**Dynein HC** (An0118, 4345 aa)	88	**150**	27	**34**	**0.18**
(Experiment 2)					
**p150**	63	**88**	12	**12**	**0.16**
**p50**	20	**34**	10	**11**	**0.37**
**Arp1**	22	**56**	11	**12**	**0.24**
**Arp11**	17	**32**	18	**28**	**1**
**p62**	17	**22**	14	**19**	**0.99**
**p25**	6	**8**	6	**7**	**1**
**Cap alpha**	6	**9**	2	**2**	**0.25**
**Cap beta**	7	**10**	1	**1**	**0.11**
**Dynein HC**	174	**224**	85	**91**	**0.46**
(Experiment 3)					
**p150**	103	**158**	77	**144**	**0.31**
**p50**	25	**51**	19	**66**	**0.44**
**Arp1**	20	**86**	26	**60**	**0.24**
**Arp11**	28	**45**	32	**132**	**1**
**p62**	19	**24**	27	**79**	**1.12**
**p25**	7	**10**	7	**29**	**0.99**
**Cap alpha**	9	**9**	9	**19**	**0.72**
**Cap beta**	12	**15**	7	**7**	**0.16**
**Dynein HC**	243	**479**	211	**552**	**0.39**

bioRxiv preprint doi: https://doi.org/10.1101/2024.04.19.590248; this version posted April 20, 2024. The copyright holder for this preprint (which was not certified by peer review) is the author/funder. This article is a US Government work. It is not subject to copyright under 17 USC 105 and is also made available for use under a CC0 license.

Table S3. *Aspergillus nidulans* strains used in this study

Strain	Genotype	Source
GR5	pyrG89; pyroA4; wA3	G.S. May
JZ505	ΔhookA-AfpyrG; GFP-nudA^{HC}; argB2::[argB*-alcAp::mCherry-RabA]; possibly pyrG89; possibly ΔnkuA::argB; pyroA4; wA2	(Zhang et al., 2014)
JZ788	Arp11-GFP-AfpyrG, argB2::[argB*-alcAp::mCherry-RabA]; ΔnkuA::argB; pyrG89; pantoB100; yA2	(Qiu et al., 2020)
RQ2	GFP-nudA^{HC}; argB2::[argB*-alcAp::mCherry-RabA]; ΔnkuA::argB; pyrG89; pyroA4; yA2	(Qiu et al., 2013)
RQ54	argB2::[argB*-alcAp::mCherry-RabA]; ΔnkuA::argB; pyrG89; pyroA4; wA2	(Qiu et al., 2013)
RQ294	GFP-nudA$^{R1602E, K1645E}$; argB2::[argB⁄-alcAp::mCherry-RabA]; ΔnkuA::argB, pyroA4; yA2	(Qiu et al., 2019)
XX222	GFP-nudA^{HC}; argB2::[argB*-alcAp::mCherry-RabA]; ΔnkuA::argB; pantoB100; yA2	(Zhang et al., 2014)
XY41	p25-GFP-AfpyrG; ΔnkuA::argB; pyrG89; pyroA4	(Zhang et al., 2014)
XY42	argB2::[argB*-alcAp::mCherry-RabA]; ΔnkuA::argB; pyrG89; pantoB100; yA2	(Qiu et al., 2018)
XY136	ΔvezA-AfpyrG; GFP-nudA^{HC}; argB2::[argB*-alcAp::mCherry-RabA]; pyrG89; ΔnkuA::argB; pyroA4; yA2	(Yao et al., 2015)
XY163	vezA-GFP-AfpyrG; argB2::[argB*-alcAp::mCherry-RabA]; pyrG89; ΔnkuA::argB pyroA4, wA2	(Yao et al., 2015)
XY167	ΔTM-vezA-GFP-AfpyrG; argB2::[argB*- alcA(p)::mCherry-RabA; ΔnkuA::argB; pyrG89; pyroA4; wA2	(Yao et al., 2015)
JZ704	ΔvezA-AfpyrG; p150-GFP-AfpyrG; argB2::[argB*-alcAp::mCherry-RabA]; pyrG89; ΔnkuA::argB	This work
JZ706	p150-GFP-AfpyrG; argB2::[argB*-alcAp::mCherry-RabA]; pyrG89; ΔnkuA::argB	This work
JZ790	p62-GFP-AfpyrG, argB2::[argB*-alcAp::mCherry-RabA]; ΔnkuA::argB; pyrG89; pantoB100; yA2	This work
JZ872	gpdA-ΔTM-vezA-GFP-AfpyrG; argB2::[argB*-alcAp::mCherry-RabA]; pyrG89; ΔnkuA::argB; pantoB100; yA2	This work
JZ942	Arp11-GFP-AfpyrG; ΔvezA-AfpyrG; argB2::[argB*-alcAp::mCherry-rabA]; ΔnkuA::argB; pyrG89; yA2	This work
JZ968	p150-GFP-AfpyrG; ΔvezA-AfpyrG; ΔhookA-AfpyrG; argB2::[argB*-alcAp::mCherry-RabA]; ΔnkuA::argB; pyrG89	This work
JZ971	Arp11-GFP-AfpyrG; ΔvezA-AfpyrG; ΔhookA-AfpyrG; argB2::[argB*-alcAp::mCherry-RabA]; ΔnkuA::argB; pyrG89	This work
JZ972	p62-GFP-AfpyrG; ΔvezA-AfpyrG; ΔhookA-AfpyrG; argB2::[argB*-alcAp::mCherry-RabA]; ΔnkuA::argB; pyrG89; yA2	This work
JZ973	p25-GFP-AfpyrG; ΔhookA-AfpyrG; argB2::[argB*-alcAp::mCherry-RabA]; ΔnkuA::argB; pyrG89; yA2	This work
JZ974	p25-GFP-AfpyrG; ΔvezA-AfpyrG; argB2::[argB*-alcAp::mCherry-RabA]; ΔnkuA::argB; pyrG89	This work
JZ975	p25-GFP-AfpyrG; ΔvezA-AfpyrG; ΔhookA-AfpyrG; argB2::[argB*-alcAp::mCherry-RabA]; ΔnkuA::argB; pyrG89	This work
JZ976	p62-GFP-AfpyrG; ΔhookA-AfpyrG; argB2::[argB*-alcAp::mCherry-	This work

bioRxiv preprint doi: https://doi.org/10.1101/2024.04.19.590248; this version posted April 20, 2024. The copyright holder for this preprint (which was not certified by peer review) is the author/funder. This article is a US Government work. It is not subject to copyright under 17 USC 105 and is also made available for use under a CC0 license.

	RabA]; ΔnkuA::argB; pyrG89; wA2	
JZ981	p62-GFP-AfpyrG; ΔvezA-AfpyrG; argB2::[argB*-alcAp::mCherry-RabA]; ΔnkuA::argB; pyrG89	This work
JZ992	p150-GFP-AfpyrG; p25-S-AfpyrG; argB2::[argB*- alcA(p)::mCherry-RabA]; ΔnkuA::argB; pyrG89	This work
JZ996	p150-GFP-AfpyrG; ΔvezA-AfpyrG; p25-S-AfpyrG; ΔnkuA::argB; pyrG89	This work
JZ1018	p25-S-AfpyrG; gpdA-ΔTM-vezA-GFP; argB2::[argB*-alcA(p)::mCherry-RabA]; ΔnkuA::argB; pyrG89; wA2	This work
JZ1025	alcA-Arp11; argB2::[argB*- alcA(p)::mCherry-RabA]; ΔnkuA::argB, pyrG89; pantoB100; yA2	This work
JZ1026	alcA-Arp1; argB2::[argB*- alcA(p)::mCherry-RabA]; ΔnkuA::argB; pyrG89; pantoB100; yA2	This work
JZ1034	alcA-p150; argB2::[argB*- alcA(p)::mCherry-RabA], ΔnkuA::argB; pyrG89; pantoB100; yA2	This work
JZ1057	vezA$^{\Delta 563\text{-}615}$-GFP-AfpyrG; argB2::[argB*-alcAp::mCherry-rabA]; ΔnkuA::argB ; pyrG89, pantoB100, yA2	This work
JZ1083	gpdA-ΔTM-vezA-GFP; alcA-p50; argB2::[argB*- alcA(p)::mCherry-RabA]; ΔnkuA::argB; pyrG89; yA2	This work
JZ1084	gpdA-ΔTM-vezA-GFP; alcA-Arp1; p25-S-AfpyrG; argB2::[argB*-alcA(p)::mCherry-RabA]; ΔnkuA::argB; pyrG89; yA2	This work
JZ1085	p25-GFP-AfpyrG; alcA-p50; argB2::[argB*-alcAp::mCherry-rabA]; ΔnkuA::argB; pyrG89; yA2	This work
JZ1095	p50-GFP-AfpyrG; ΔvezA-AfpyrG; argB2::[argB*-alcAp::mCherry-rabA]; ΔnkuA::argB; pyrG89; wA2	This work
JZ1108	vezA$^{\Delta 1\text{-}20}$-GFP-AfpyrG; argB2::[argB*-alcAp::mCherry-rabA]; ΔnkuA::argB; pyrG89; pyroA4; wA2	This work
JZ1117	p25-GFP-AfpyrG; p50-S-AfpyrG; argB2::[argB*-alcAp::mCherry-rabA]; ΔnkuA::argB; pyrG89	This work
JZ1121	p25-GFP-AfpyrG; alcA-p150; p50-S-AfpyrG; argB2::[argB*-alcAp::mCherry-rabA]; ΔnkuA::argB; pyrG89; yA2	This work
JZ1122	Arp11-GFP-AfpyrG; p50-S-AfpyrG, argB2::[argB*-alcAp::mCherry-rabA]; ΔnkuA::argB; pyrG89	This work
JZ1123	Arp11-GFP-AfpyrG; p50-S-AfpyrG; ΔvezA-AfpyrG; argB2::[argB*-alcAp::mCherry-rabA]; ΔnkuA::argB; pyrG89	This work
JZ1125	p50-GFP-AfpyrG; alcA-Arp1; argB2::[argB*-alcAp::mCherry-rabA]; ΔnkuA::argB; pyrG89; yA2	This work
RQ70	p25-S-AfpyrG; argB2::[argB*-alcAp::mCherry-rabA]; ΔnkuA::argB; pyrG89; pyroA4; wA2	This work
SX1	p50-S-AfpyrG, argB2::[argB*-alcAp::mCherry-rabA]; ΔnkuA::argB; pyrG89; pantoB100; yA2	This work
SX2	p50-GFP-AfpyrG, argB2::[argB*-alcAp::mCherry-rabA]; ΔnkuA::argB; pyrG89; pyroA4; wA2	This work
XX365	ΔvezA-AfpyrG; GFP-histone H1 (hhoA-GFP-Afribo); argB2::[argB*-alcAp::mCherry-RabA]; possibly pyrG89; possibly ΔnkuA::argB, wA2	This work
XX367	GFP-histone H1 (hhoA-GFP-Afribo); argB2::[argB*-alcAp::mCherry-RabA]; possibly pyrG89; possibly ΔnkuA::argB	This work
XX370	p150-GFP-AfpyrG; argB2::[argB*-alcAp::mCherry-RabA]	This work
XX371	ΔvezA-AfpyrG; p150-GFP-AfpyrG; argB2::[argB*-alcAp::mCherry-	This work

bioRxiv preprint doi: https://doi.org/10.1101/2024.04.19.590248; this version posted April 20, 2024. The copyright holder for this preprint (which was not certified by peer review) is the author/funder. This article is a US Government work. It is not subject to copyright under 17 USC 105 and is also made available for use under a CC0 license.

	RabA]	
XX374	*ΔvezA-AfpyrG; argB2::[argB⁄-alcAp::mCherry-RabA]; ΔnkuA::argB, pyrG89; pantoB100*	This work
XX518	*ΔvezA-AfpyrG;* GFP-*nudA*^{HC}*; gpdA-Δ*C-*hookA*-S-*AfpyrG; pyrG89; yA*1, *ΔnkuA::argB*	This work
XX618	GFP-*nudA*$^{R1602E, K1645E}$; *ΔvezA-AfpyrG; argB2::[argB⁄-alcAp::mCherry-RabA]; ΔnkuA::argB, pyrG89;*(RQ294x JZ698)	This work
XX721	p150-GFP-*AfpryG*; *ΔhookA-AfpryG*; *argB2::[argB⁄-alcAp::mCherry-RabA]*	This work
XX723	p150-GFP-*AfpryG*; *ΔhookA-AfpryG*; *ΔvezA-AfpyrG; argB2::[argB⁄-alcAp::mCherry-RabA]*	This work
XX760	Arp11-GFP-*AfpryG*; *ΔhookA-AfpryG*; *argB2::[argB⁄-alcAp::mCherry-RabA]*	This work
XX762	Arp11-GFP-*AfpryG*; *ΔhookA-AfpryG*; *ΔvezA-AfpyrG; argB2::[argB⁄-alcAp::mCherry-RabA]*	This work
XX1017	p50-GFP-afpryG; *ΔhookA-AfpryG*; *argB2::[argB⁄-alcAp::mCherry-RabA]; wA2*	This work
XX1018	p50-GFP-afpryG; *ΔhookA-AfpryG*; *ΔvezA-AfpyrG; argB2::[argB⁄-alcAp::mCherry-RabA]; wA2*	This work
